# Cadmium Accumulation and Pathological Alterations in the Midgut Gland of Terrestrial Snail *Helix pomatia L.* from a Zinc Smelter Area: Role of Soil pH

**DOI:** 10.1007/s00128-016-1748-0

**Published:** 2016-02-11

**Authors:** Tadeusz Włostowski, Paweł Kozłowski, Barbara Łaszkiewicz-Tiszczenko, Ewa Oleńska

**Affiliations:** Institute of Biology, University of Białystok, Ciołkowskiego 1J, 15-245 Białystok, Poland

**Keywords:** Cadmium, Snails, Midgut gland, Metallothionein, Lipid peroxidation, Lipofuscin

## Abstract

The purpose of this study was to determine whether cadmium (Cd) accumulation and toxicity in the midgut gland of *Helix pomatia* snails living in a Cd-contaminated area were related to soil pH. Toxic responses in the midgut gland (i.e., increased vacuolization and lipid peroxidation) occurred in *H. pomatia* snails exhibiting the highest Cd levels in the gland (265–274 µg/g dry wt) and living on acidic soil (pH 5.3–5.5), while no toxicity was observed in snails accumulating less Cd (90 µg/g) and ranging on neutral soil (pH 7.0), despite the fact that total soil Cd was similar in the two cases. The accumulation of Cd in the gland was directly related to the water extractable Cd in soil, which in turn correlated inversely with soil pH, indicating that this factor had a significant effect on tissue Cd. It appeared further that the occurrence of Cd toxicity was associated with low levels of metallothionein in the gland of snails ranging on acidic soil.

Cadmium (Cd) is an important toxic metal occurring in the environment naturally and as a pollutant emanating mainly from industrial sources such as smelting and refining of zinc and lead ores (Pan et al. 2010). Environmental Cd has been proven to induce damage primarily to the kidneys of humans (Järup and Akesson 2009), and wildlife such as ptarmigan *Lagopus leucurus* (Larison et al. 2000), roe deer *Capreolus capreolus* (Beiglböck et al. 2002), and magpies *Pica pica* (Włostowski et al. 2010). Under laboratory conditions, Cd has also been shown to produce toxicity in various tissues of molluscs, including *Helix pomatia* snails (Chabicovsky et al. 2004; Hödl et al. 2010; Amachree et al. 2013; Sheir et al. 2013). The studies demonstrated that pathological changes typical for Cd toxicity, specifically in the midgut gland, include an increased number of excretory cells with residual bodies (lipofuscin granules), programmed cell death, the disruption of mitochondrial membranes, and inflammation (Chabicovsky et al. 2004; Hödl et al. 2010; Amachree et al. 2013; Sheir et al. 2013). These alterations are thought to occur at Cd concentrations exceeding the capacity of metallothionein (MT), a low-molecular weight Cd-binding protein which is responsible for detoxification of the metal in the midgut gland of *H. pomatia* and other species (Dallinger et al. 1997; Chabicovsky et al. 2004; Hödl et al. 2010; Baurand et al. 2015). The non-MT-bound Cd ions are believed to generate reactive oxygen species and peroxidation of membrane lipids, thereby producing toxicity (Roesijadi et al. 1997; Liu et al. 2009; Amachree et al. 2013).

It is known that elevated levels of Cd in the midgut gland of *H. pomatia* can cause toxicity (Chabicovsky et al. 2004; Hödl et al. 2010), and the level of Cd accumulation in the midgut gland of terrestrial snails has been shown to be related mainly to its total concentration in the food (plant and soil), and exposure time (Laskowski and Hopkin 1996; Dallinger et al. 2004; Hödl et al. 2010). The laboratory and field studies also revealed that soil pH is a very important factor influencing Cd bioavailability to various soil invertebrates, such as earthworms (Ma et al. 1983), snails (Pauget et al. 2012), and collembolans (Ardestani and van Gestel 2013). So far, however, little is known about the role of soil pH in the accumulation and toxicity of Cd in terrestrial snails, such as *H. pomatia* ranging freely in a Cd-contaminated area.

Therefore, the present work was designed to determine whether Cd accumulation and toxicity (if any) in the midgut gland of *Helix pomatia* snails, free living in residential areas located around a zinc smelter, were related to soil pH. The toxicity was evaluated by assessing in the gland a number of excretory cells with lipofuscin granules (Hödl et al. 2010), and lipid peroxidation (Amachree et al. 2013). The concentration of MT that is linked to a protective effect against Cd toxicity was also examined.

## Materials and Methods

**Fig. 1:**
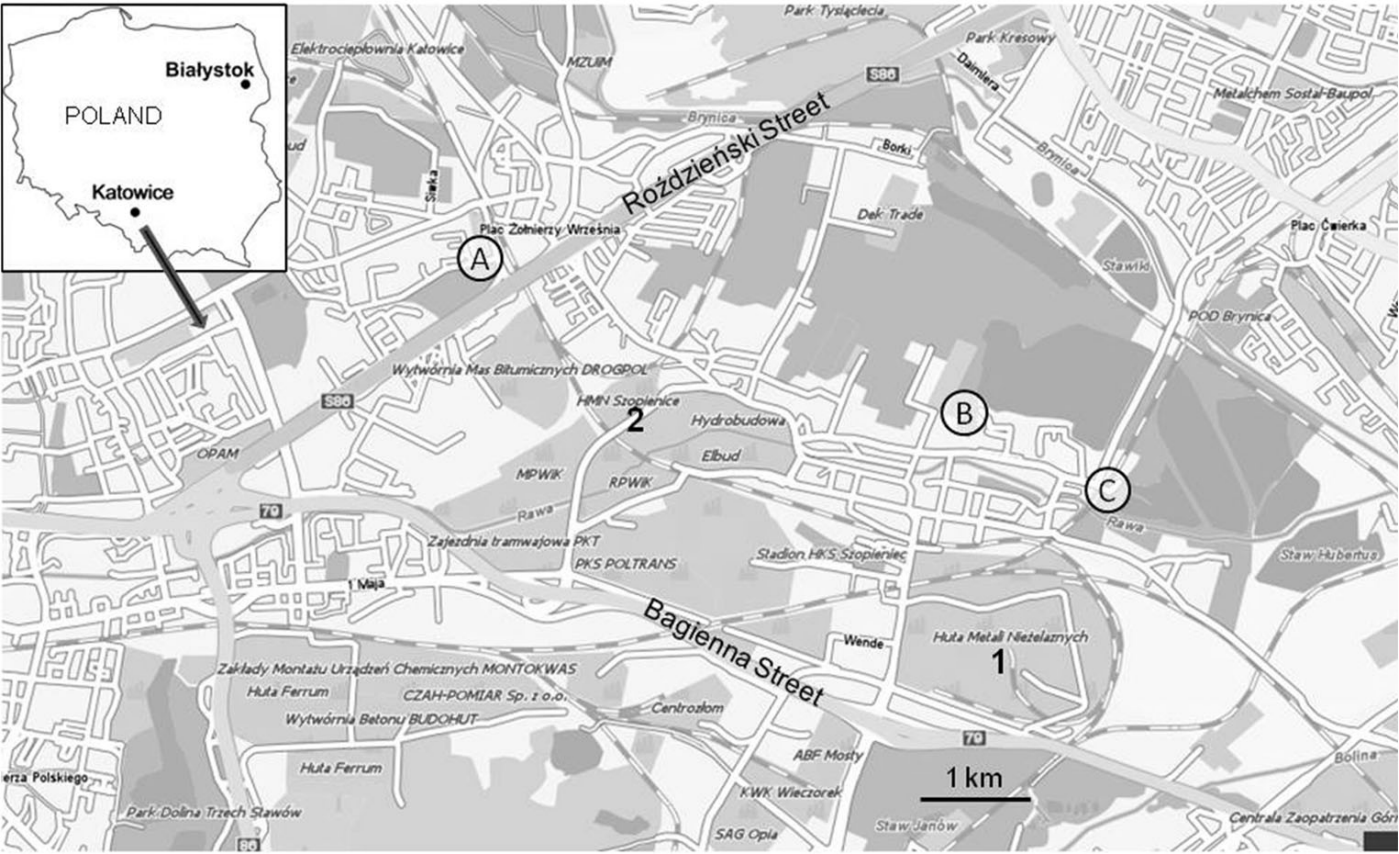
Map showing location of a reference (Białystok) and zinc smelter area (Katowice). *A*, *B* and *C* indicate sampling sites in Katowice. *1* and *2* indicate locations of former zinc smelters

Ten individuals of *Helix pomatia* snails of similar body weight (16.5–18.5 g) were collected in the first week of July 2012 from each of three residential areas situated around former zinc smelters in Katowice (southern part of Poland – here referred to as Cd-contaminated sites) and from a residential area of Białystok (one of the least contaminated cities in Poland (Włostowski et al. 2014) – here referred to as a reference site) (Fig. 1). Sampling locations were pre-screened for soil Cd, as well as soil pH and organic matter which are known to affect Cd bioavailability (Pauget et al. 2012). For this purpose, three composite samples of topsoil (0–5 cm) were taken from each site. As can be seen in Table 1, the total content of Cd and organic matter in the topsoil was similar in the three Cd-contaminated sites, but soil pH was significantly lower at sites B and C than at site A.

**Table 1:** Concentrations of total Cd, water extractable Cd, pH and organic matter content of soil from a reference and zinc smelter area (Katowice)

	Białystok (reference site)	Katowice A	Katowice B	Katowice C
Total soil Cd (µg/kg)	100 ± 30^a^	2100 ± 250^b^	1860 ± 300^b^	1960 ± 350^b^
Water extractable Cd (µg/kg)	1.0 ± 0.20^a^	10.00 ± 2.10^b^	48.0 ± 8.0^c^	55.0 ± 5.0^c^
Soil pH	7.25 ± 0.10^a^	7.01 ± 0.05^a^	5.50 ± 0.05^b^	5.30 ± 0.10^b^
Organic matter (%)	5.1 ± 0.7^a^	5.5 ± 1.0^a^	6.1 ± 0.8^a^	6.0 ± 0.9^a^

Data are presented as mean ± SD for n = 3. Means in the same row marked with a different superscript letter are significantly different (*p* < 0.05) (ANOVA and Duncan’s multiple range test)

In the laboratory, all the snails were anesthetized (5 % ethanol), decapitated and the midgut gland was removed and divided into three portions. One portion (about 200 mg wet weight) was dried at 60°C for 48 h and used for determination of Cd and water (to calculate a dry weight conversion factor). Another one was fixed in 4 % formaldehyde for histological examination. The third portion was frozen and kept at −80°C until analysis of MT and lipid peroxidation. After thawing, a portion (about 250 mg) of this organ was transferred to 1.0 mL chilled 0.25 M sucrose and homogenized with a Teflon pestle in a glass homogenizer. An aliquot (0.2 mL) was taken for determination of lipid peroxidation. The remaining homogenate was centrifuged at 20,000×*g* for 20 min at 4°C, and the resulting supernatant was removed for MT assay. The data were expressed on a dry weight basis, using dry weight conversion factors.

Cd content in the midgut gland of all *H. pomatia* snails was determined as follows: a portion of the organ (60–70 mg dry weight) was digested at 200°C for 40 min in a mixture of redistilled nitric acid (70 %) (Sigma-Aldrich) (2.5 mL), 30 % H_2_O_2_ (Sigma-Aldrich) (0.25 mL) and deionized water (2.25 mL), using a Mars 6 microwave oven (CEM Corporation, Matthews, NC, USA); Cd as well as zinc (Zn), copper (Cu) and lead (Pb) analyses of these solutions were carried out by electrothermal atomic absorption spectrometry (AAS), using a Thermo iCE 3400 instrument with Zeeman correction (Thermo Electron Manufacturing Ltd, Cambridge, UK). Quality assurance procedures included the analysis of reagent blanks and standard reference material (Bovine liver 1577c – National Institute of Standards and Technology, Gaithersburg, MD). The precision expressed as relative standard deviation (RSD) of 10 measurements of the same sample was 5 %–8 %, and the recoveries of Cd, Zn, Cu and Pb were 88 %–98 %.

In the case of soil Cd two different extractions were performed. To measure total Cd concentration, 0.2 g of soil (passed through a 1 mm sieve) was extracted at 180°C for 15 min with concentrated nitric acid (4.5 mL) and HF (1.5 mL) (Method 3052, US EPA 1996) in a Mars 6 microwave oven. Samples of Montana II soil 2711a (NIST) were also analyzed in an identical manner to check accuracy of the method. The recovery of Cd was 90 %–95 %. To measure available Cd concentration, 0.5 g of the soil was extracted with 2.0 mL deionized water for 24 h. The soil suspensions were centrifuged at 30,000×*g* for 30 min and the supernatants were analyzed for Cd by electrothermal AAS. Soil pH was determined in soil suspensions (1:2.5 soil to water ratio) incubated for 24 h, using a pH meter. Organic matter content was measured from loss-on-ignition (600°C, 2 h).

Metallothionein in the midgut gland was determined by a Cd-saturation method (Włostowski et al. 2010). Briefly, a 0.1 mL sample was incubated for 10 min with 1.0 mL Tris–HCl buffer containing 1.0 µg Cd/mL. To remove non-MT-bound Cd, bovine hemoglobin was added and the sample was heated at 95°C and centrifuged. Cd bound to MT was determined by AAS. MT was expressed in µg Cd bound to the protein/g tissue.

Lipid peroxidation was assessed by measuring malondialdehyde (MDA) formation, using the thiobarbituric acid (TBA) assay (Ohkawa et al. 1979). Briefly, to 0.2 mL of the tissue homogenate, 0.2 mL of 8.1 % sodium dodecyl sulfate, 1.5 mL of 20 % acetic acid, 1.5 mL of 0.8 % TBA and 0.6 mL of water were added. The reaction mixture was heated at 95°C for 1 h. After cooling, 1.0 mL of water and 5.0 mL of butanol/pirydyne mixture were added. Absorbance of the organic phase was determined at 532 nm. The results were expressed as TBARS (nmol/g).

The fixed portions of the midgut gland were dehydrated in ethanol and xylene, embedded in paraffin, cut into 5 µm sections (Leica microtome), and stained with hematoxylin and eosin for microscopic examination (Zawistowski 1975). The number of excretory cells per tubule cross-section was estimated.

Data were expressed as mean ± SD. They were analyzed by one-way analysis of variance (ANOVA) followed by the Duncan’s multiple range test (when required, the variables were normalized using log_**10**_ transformation). Differences at *p* < 0.05 were considered statistically significant. The simple regression analysis was used to examine the relationship between Cd accumulation in the midgut gland and water extractable Cd in soil, and between the water extractable Cd and soil pH. All the statistical analyses were performed using IBM SPSS Statistics 21 program (IBM Corporation, Somers, NY, USA).

## Results and Discussion

**Fig. 2:**
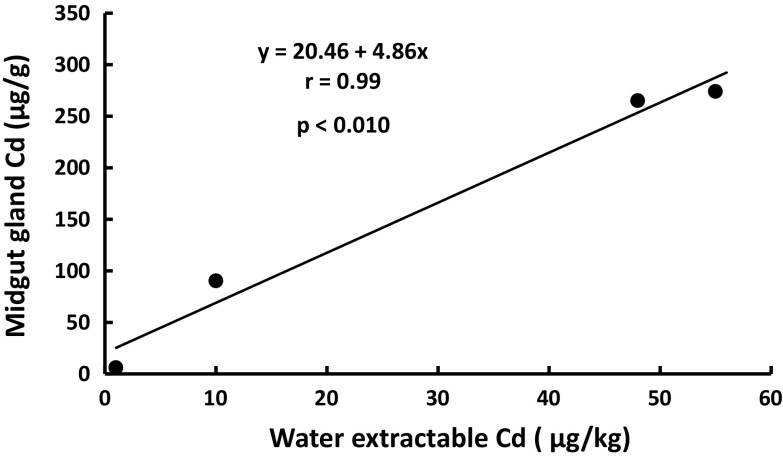
The relationship of water extractable Cd in soil to the midgut gland Cd in *Helix pomatia* snails under study (mean values from Tables 1 and 2 were compared)

**Fig. 3:**
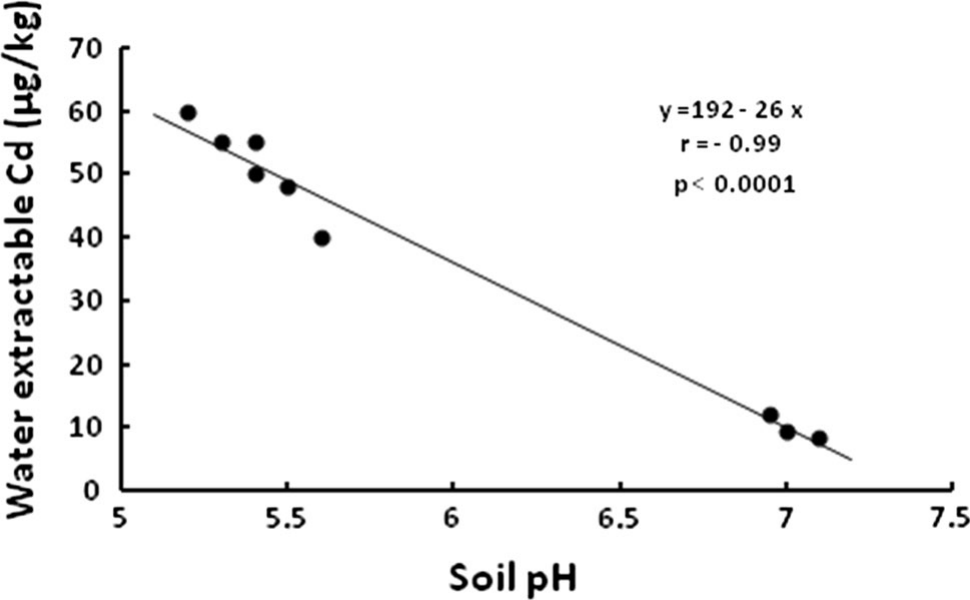
The relationship of soil pH to the water extractable Cd in soil from a zinc smelter area. Soil samples (n = 3) from each of the three Cd-contaminated sites were used in the analysis

All *H. pomatia* snails caught in Cd-contaminated sites (Katowice A, B and C) and in a reference site (Białystok) had normal gross morphology; for the purposes of this study only animals of similar body mass were selected, to avoid the possibility of confounding effects on Cd accumulation due to different body masses (Dallinger 1994). Although total Cd concentration in soil was similar in the three contaminated sites (Table 1), the accumulation of Cd in the midgut gland of *H. pomatia* snails differed significantly (*p* < 0.01) amongst the sites (Table 2); the snails from sites B and C accumulated threefold higher amounts of Cd than those inhabiting site A, and 50-fold higher than those living in a reference site. As can be seen in Tables 1 and 2, and Fig. 2, the accumulation of Cd in the gland correlated significantly with the water extractable Cd in soil, suggesting that the available fraction of this metal could account for the observed differences. Furthermore, the simple regression analysis revealed that in the contaminated area the water extractable Cd correlated inversely with soil pH (Fig. 3; Table 1), indicating that this factor could have a significant effect on Cd accumulation in *H. pomatia* snails.

**Table 2:** Body and organ weights, cadmium and metallothionein levels, lipid peroxidation (TBARS) and number of excretory cells, and zinc, copper and lead concentrations in the midgut gland of *Helix pomatia* snails from a reference and zinc smelter area (Katowice)

	Białystok (Reference site)	Katowice A	Katowice B	Katowice C
Body weight (g)	17.0 ± 0.8^a^	17.2 ± 1.0^a^	17.3 ± 0.6^a^	17.5 ± 0.5^a^
Midgut gland weight (g)	1.45 ± 0.18^a^	1.52 ± 0.15^a^	1.51 ± 0.13^a^	1.56 ± 0.14^a^
Cadmium (µg/g)	6.10 ± 2.40^a^	90.3 ± 20.4^b^	265 ± 70^c^	274 ± 90^c^
Metallothionein (µg Cd/g)	8.00 ± 2.50^a^	102 ± 17.0^b^	200 ± 45^c^	210 ± 50^c^
TBARS (nmol/g)	180 ± 27^a^	210 ± 26^a^	300 ± 25^b^	338 ± 32^b^
Excretory cells/tubule	3.20 ± 0.50^a^	4.05 ± 0.55^a^	6.90 ± 0.50^b^	7.30 ± 0.45^b^
Zinc (µg/g)	305 ± 200^a^	7715 ± 1500^b^	9675 ± 2500^b^	9800 ± 1700^b^
Copper (µg/g)	18.8 ± 5.3^a^	165 ± 52^b^	140 ± 43^b^	130 ± 45^b^
Lead (µg/g)	7.38 ± 2.52^a^	34.9 ± 3.5^b^	18.1 ± 4.1^c^	15.1 ± 3.2^c^

Data are presented as mean ± SD for n = 10. Means in the same row marked with a different superscript letter are significantly different (*p* < 0.05) (ANOVA and Duncan’s multiple range test)

The midgut gland was also analyzed for MT content, a well-known protective molecule (Table 2). In general, the Cd-binding capacity of MT in this organ followed a pattern similar to that of Cd concentration; however, the capacity of MT exceeded the total concentration of Cd only in snails from a reference site and Cd-contaminated site A, but Cd level exceeded MT capacity (by 65 µg/g) in animals ranging in sites B and C (Table 2).

**Fig. 4:**
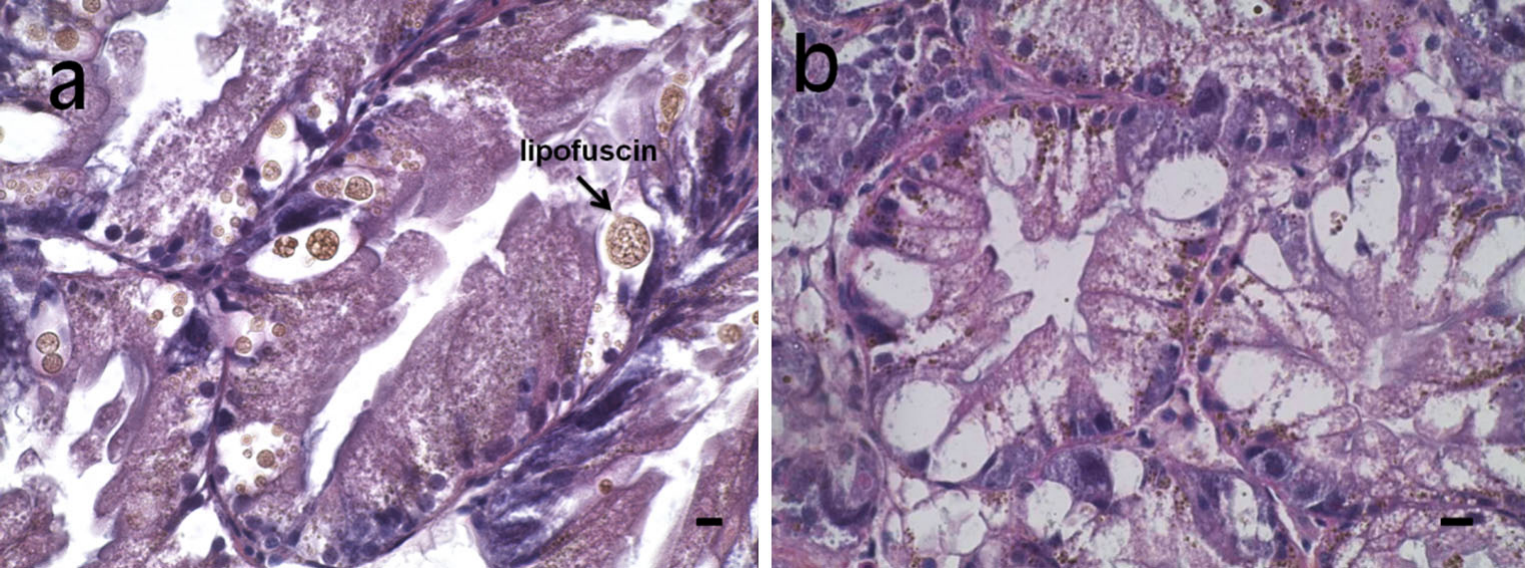
Representative photomicrograph of a midgut gland section from the *Helix pomatia* snails (**a**) ranging in a reference (Białystok) and zinc smelter area – Katowice A, and **b** living in a zinc smelter area – Katowice B und C. Note the presence (**a**) and absence (**b**) of lipofuscin granules within large vacuoles of excretory cells. *Scale bar* 20 µm

It has previously been demonstrated in laboratory conditions that Cd intoxication of *H. pomatia* snails increases the formation of lipofuscin granules (residual bodies) in the excretory cells of the midgut gland epithelium (Hödl et al. 2010). In the present study, *H. pomatia* snails with the highest Cd concentration in the gland (sites B and C) exhibited an increased number of excretory cells in the epithelium, but without residual bodies; in contrast, the reference animals and those living in Cd-contaminated site A had significantly fewer excretory cells, but containing lipofuscin granules (Fig. 4, Table 2). Likewise, lipid peroxidation (TBARS) in the midgut gland of snails from sites B and C was significantly higher than that in animals from the two other sites (Table 2).

The present study demonstrated that a relatively low soil pH substantially increases Cd accumulation in the midgut gland of *H. pomatia* snails free ranging in the metal contaminated area. This effect was probably indirect, through the consumption of Cd-contaminated plants. These animals feed mainly on plants (Dallinger et al. 2004; Pauget et al. 2015), and a low soil pH increases Cd solubility and availability, particularly for uptake by plants (Erikson et al. 1996). It is also possible that the ingestion of soil, and Cd uptake via the foot epithelium could contribute, at least to some degree, to this effect (Coeurdassier et al. 2002; Pauget et al. 2015).

The involvement of soil pH in the accumulation of Cd in tissues of other animals has also been demonstrated. For instance, Pauget et al. (2012) have found that an acid soil pH increases Cd accumulation kinetics in the snail *Cantareus aspersus*. Also, an inverse correlation between soil pH and Cd accumulation in the liver and kidneys of big and small mammals has been documented (Włostowski et al. 2006). Thus, a low soil pH appears to increase tissue Cd levels in various animals, thereby raising the risk of toxicity. Indeed, the toxicity in the midgut gland occurred in *H. pomatia* snails exhibiting the highest Cd levels (265–274 µg/g) and living on acidic soil, while no toxicity was observed in snails accumulating less Cd (90 µg/g = 0.8 µmol/g) and ranging on neutral soil, despite the fact that total soil Cd concentration was similar in the two cases (Table 2).

The results of the present study are in agreement with those of Hödl et al. (2010), who demonstrated in laboratory conditions that pathological changes in the midgut gland of *H. pomatia*, such as an increased number of excretory cells with lipofuscin granules, occurred only at Cd levels that exceeded a threshold of 0.8 µmol/g. These authors also revealed that MT protein became fully occupied by Cd ions when their concentration exceeded the threshold, and further Cd loading led to the interactions with cellular macromolecules and enzymes, thereby inducing toxicity. It is therefore likely that the non-MT-bound Cd ions were responsible for the induction of oxidative stress (TBARS) and histopathological alterations in the midgut gland of *H. pomatia* snails examined in the present study. Importantly, Cd-induced oxidative stress is thought to lead to irreversible degradation of macromolecules and the degradation products (lipofuscin) are accumulated in residual bodies (Hödl et al. 2010). Thus, the number of residual bodies in the excretory cells should increase rather than decrease, as was observed in the present work (Fig. 4).

Although the precise mechanism for the observed decline in residual bodies is unknown, this phenomenon may be explained, at least partly, by the fact that at high Cd tissue levels the rate of lipofuscin granule release by excretory cells into the tubule lumen may be enhanced (Hödl et al. 2010). Furthermore, *H. pomatia* snails from the zinc smelter areas were also chronically exposed to other metals (Zn, Cu, Pb) which could contribute to these changes (Zaldibar et al. 2008). However, in contrast to Cd, the concentrations of Zn and Cu were similar in the snails from three contaminated sites in Katowice, and the accumulation of Pb was significantly higher in animals from site A than sites B and C (Table 2). Thus, these metals were probably not involved solely, but could potentiate Cd toxicity in the midgut gland. It is also possible that the presence or absence of lipofuscin granules in excretory cells may have been influenced by the diurnal activity of snails or preceding estivation due to dry weather conditions. Further studies are needed to determine why the histological picture of the midgut gland epithelium of free-living *H. pomatia* snails is different from that observed in snails accumulating comparable Cd concentrations under laboratory conditions (Hödl et al. 2010).

In conclusion, the results of the present study showed that a relatively low soil pH dramatically increased the accumulation of Cd in the midgut gland of *H. pomatia* snails free ranging in a zinc smelter area. The accumulated Cd appeared to induce toxicity in the gland, with Cd ions not bound to MT likely being responsible for these observed effects.
